# Walking School Bus Programs: Implementation Factors, Implementation Outcomes, and Student Outcomes, 2017–2018

**DOI:** 10.5888/pcd17.200061

**Published:** 2020-10-15

**Authors:** Jordan A. Carlson, Chelsea Steel, Carolina M. Bejarano, Marshall T. Beauchamp, Ann M. Davis, James F. Sallis, Jon Kerner, Ross Brownson, Sara Zimmerman

**Affiliations:** 1 Center for Children’s Healthy Lifestyles and Nutrition, Children’s Mercy Hospital, Kansas City, Missouri; 2 Clinical Child Psychology Program, University of Kansas, Lawrence, Kansas; 3 Clinical Psychology Program, University of Missouri, Kansas City, Missouri; 4 Department of Pediatrics, University of Kansas Medical Center, Kansas City, Kansas; 5 Department of Family Medicine and Public Health, University of California San Diego, La Jolla, California; 6 Mary MacKillop Institute for Health Research, Australian Catholic University, Melbourne, Australia; 7 Canadian Partnership Against Cancer, Toronto Ontario, Canada; 8 Prevention Research Center in St. Louis, Brown School, Washington University in St. Louis, St. Louis, Missouri; 9 Division of Public Health Sciences, Washington University School of Medicine, Washington University in St. Louis, St. Louis, Missouri; 10 Safe Routes Partnership, Berkeley, California

## Abstract

**Purpose and Objectives:**

Walking school bus programs increase children’s physical activity through active travel to school; however, research to inform large-scale implementation of such programs is limited. We investigated contextual factors, implementation outcomes, and student outcomes in existing walking school bus programs in the United States and internationally.

**Intervention Approach:**

Walking school bus programs involve a group of children walking to school together with an adult leader. On the trip to school, these adults provide social support, address potential traffic and interpersonal safety, and serve as role models to the children while children increase their physical activity levels.

**Evaluation Methods:**

We conducted surveys with existing walking school bus programs identified through internet searches, referrals, and relevant email listservs. Leaders from 184 programs that operated at least 1 trip per week completed the survey. We used regression analyses to compare differences in contextual factors by area income and location, associations between contextual factors and implementation outcomes, and associations between implementation outcomes and student outcomes.

**Results:**

Walking school bus programs in low-income communities had more route leaders and engaged in more active travel to school-related activities of being sustained than those in higher income. Programs that had no external funding, multiple route leaders, and coordination by a school or district staff member (as opposed to a parent) had greater student participation than other programs. Providing more trips than other programs per week was associated with reduced tardiness, reduced bullying, and improved neighborhood walkability. The greatest barriers to implementation were recruiting and maintaining students and identifying and maintaining route leaders.

**Implications for Public Health:**

Walking school bus programs can be implemented successfully in many contexts using various models. The involvement of several people in leadership roles is critical for sustainability. Evidence-based implementation strategies that overcome barriers can improve reach, implementation, and sustainability of walking school bus programs and can increase children’s physical activity.

**Summary**

**What is already known on this topic?**

Walking school bus programs increase children’s physical activity through active travel to school; however, research to inform large-scale implementation of such programs is limited.

**What is added by this report?**

We investigated implementation contextual factors, implementation outcomes, and student outcomes in existing walking school bus programs in the United States and internationally.

**What are the implications for public health practice?**

Ours is the first study to investigate implementation factors in a large sample of walking school bus programs. To improve the reach and population effects in different models of walking school bus programs, effective strategies for engaging students, parents, and other community members are needed.

## Introduction

The Community Guide recommends increasing the proportion of children who walk to school (1). Children who regularly walk to school exercise an average of 15 to 20 more minutes per day for total physical activity, have a lower body mass index (BMI) and lower risk for chronic diseases than their counterparts (2–7). Although walking to school supports healthy development, learning, and cognition (8–14), less than 20% of elementary-aged children walk to school in the United States (15–19). Walking to school is an important intervention strategy for improving public health, because large numbers of children can be reached without disrupting time in school; however, greater uptake of effective interventions is needed to support increases in walking to school.

In the United States, the Safe Routes to School (SRTS) movement has gained substantial momentum over the past 2 decades in support of walking to school (20). SRTS programs often involve collaborations between schools, community organizations, and city engineers to provide strategies, including infrastructure and psychosocial supports, and have shown effective for increasing rates of walking to school (15,21). A promising noninfrastructure intervention that has been included in some SRTS efforts is the walking school bus. Walking school bus programs involve a group of children walking to school together with an adult leader. On the trip to school, these adults provide social support, address potential traffic and interpersonal safety, and serve as role models to the children. Walking school bus programs have been adopted in an estimated 6% of US elementary schools and in schools of other countries (22–25), providing evidence for increasing overall physical activity (26–28). Limited research is available, however, to guide real-world implementation, which is important to support existing efforts and increase adoption and scalability of more walking school bus programs (29).

Although the concept of a walking school bus is simple, the success and sustainability of a walking school bus program are likely influenced by multiple contextual factors. Contextual factors are elements that surround an implementation effort that affect the extent to which the intervention is put into practice as intended and is successful in promoting intended outcomes in real-world settings (30,31). Several frameworks have been developed to organize contextual factors (10,30,32,33) and outcomes, including fidelity, reach and penetration, and sustainability (34). To support improved success of walking school bus programs, a crucial next step in research is to better understand how various contextual factors, such as personal supports and resources, relate to key implementation outcomes. These efforts could help determine strategies to overcome barriers and improve facilitators to implementation.

## Purpose and Objectives

We aimed to describe contextual factors and outcomes within existing regularly operating (ie, at least weekly) walking school bus programs in the United States and other countries, assess associations between contextual factors and implementation outcomes, and assess associations between implementation outcomes and student outcomes (eg, tardiness, classroom behavior). Walking school bus programs in the United States were our primary focus because of their low prevalence (22) and the growing national SRTS movement. Efforts were made, however, to include programs from other countries because they might use unique strategies and processes that can inform the success of programs in the United States.

## Intervention Approach

Walking school bus programs involve a group of children walking to school together with an adult leader. Routes are created between students’ homes and the school, and students are picked up along the walk to school. Walking school bus programs can be operated by school staff, parents, and community members. The walks can occur to or from school, daily or less frequently.

## Evaluation Methods

We surveyed key informants from existing walking school bus programs. All walking school bus routes for a school were considered a single program, and some key informants, such as staff from community organizations, led walking school bus programs at multiple schools. Existing walking school bus programs were identified through multiple sources, including internet searches, US SRTS coordinators and health departments (35), members of the International Physical Activity and the Environment Network (36), and US travel to school-related listservs. Internet searches aimed to identify both existing programs (eg, from school websites, blog posts, news articles) and health- or pedestrian-focused organizations that supported local active travel to school activities. For recruitment in the United States, 1,016 intermediary sources (eg, state SRTS coordinators) and 319 other sources believed to be directly involved in a walking school bus within the past 3 years were contacted. For recruitment outside of the United States, 289 intermediary sources (eg, community organizations) and 270 other sources believed to be directly involved in a walking school bus program from 2017 to 2018 were contacted. We were unable to determine how often an intermediary source forwarded the survey link to their network that might have included walking school bus leaders, however, making it impractical to track response rates.

Study eligibility required respondents to be a coordinator or other leader of a walking school bus or bicycle train program at 1 or more schools. Bike trains were included because organizational-level implementation was perceived to be similar between bike trains and walking school buses (eg, benefits, barriers, processes). Coordinators of programs at multiple schools could complete the survey multiple times but only once for each school. Programs with less than 1 trip per week were excluded because they were likely not frequent enough to produce health benefits and might have faced different barriers than regularly operating programs. The online survey was created in REDCap (Research electronic data capture, Vanderbilt University) (37) and sent by email to the identified contacts with the eligibility criteria stated directly at the beginning. Contacts who did not meet the eligibility criteria were asked to forward the survey to the coordinator of their program or to anyone they knew who met the criteria. All key informants provided informed consent and were offered a $25 gift card incentive. The study was approved by Children’s Mercy Hospital research ethics oversight committee.

### Measures

**Overview.** We developed a survey to capture easily assessed descriptive information and implementation characteristics. Items related to implementation outcomes and student outcomes were guided by Proctor’s model for implementation research (34,38). Items related to contextual factors were guided by implementation concepts (eg, organizational factors, barriers, facilitators) that are included in multiple frameworks and are, therefore, not specific to a single framework (30,33,39,40). A select subset, rather than all constructs from these frameworks, was included to prevent the survey from becoming overly burdensome to participants. Survey items were discussed among study team members and collaborators with experience delivering walking school bus programs until consensus was reached on items to retain and their wording. The survey was then pilot tested with 3 walking school bus leaders to inform edits to support comprehension and readability.

**Implementation outcomes.** Implementation outcomes included the number of students who regularly participated in the walking school bus (reach), number of trips per week (1–10 doses) and likelihood that the program would continue the following year (1 = extremely unlikely, 2 = somewhat unlikely, 3 = somewhat likely, 4 = extremely likely, sustainable).

**Perceived student outcomes**. Student outcomes included whether the respondent was aware of any improvements in student behavior in the classroom, reductions in tardiness, reductions in bullying, or improvements in neighborhood walkability and safety that resulted from the program (“yes,” “no,” “don’t know”). Don’t know responses were recoded as no (was not aware of improvement) for the statistical analyses. These nonhealth outcomes were assessed because of their importance to school decision makers, and a nonhealth benefits index of 0–4 was created by summing responses to the 4 items.

**Implementation contextual factors.** Contextual factors captured aspects of the built environment around the school, characteristics of the program and program coordinator, internal and external resources and supports available, and engagement activities to enhance the program (Table 1).

**Table 1:** Measures of Implementation Contextual Factors for Evaluation of 184 International Walking School Bus Programs, 2017–2018

Implementation Contextual Factor	Number of Items	Description
**Built environment**		
Environment supportiveness	3	Whether during the walk to school, students were safe from traffic, students were safe from crime, and the availability and condition of sidewalks was adequate (yes = 1; no = 0). Items were summed to create the environmental supportiveness index (0–3).
**Program coordination**		
Who program was coordinated by	1	The coordinator’s (respondent’s) usual role outside of operating the program from a list of 12 options that were grouped as school or district staff, parent, or external organization staff (eg, health department, community nonprofit).
**Route leader characteristics**		
Number of route leaders	1	The number of route leaders other than the survey respondent.
School route leader involvement	3	Whether the program had successfully involved classroom teachers, physical education teachers, or other school staff as route leaders (yes = 1; no = 0). Items were summed to create the school route leader involvement index (0–3).
External route leader involvement	4	Whether the program had successfully involved parents, college students, retired older adults, or employees from local companies or organizations as route leaders (yes = 1; no = 0). Items were summed to create the external route leader involvement index (0–4).
**Program funding**		
External	1	Whether the program was a current recipient of external grant funding (yes = 1; no = 0).
**Supports and resources**		
Personal school supports	7	Whether each of the following people provided support to the program: superintendent; principal; other administrative staff; school nurse; physical education teacher; other teachers; wellness committee (yes = 1; no = 0). Items were summed to create the school supports index (0–7).
Parent supports	2	Whether each of the following people provided support to the program: parents, parent-teacher organization (yes = 1; no= 0). Items were summed to create the school supports index (0–2).
**Support activities**		
Built environment activities	6	Whether any of the following neighborhood environment or walkability improvements had been made to support the program: sidewalk improvements, crosswalk or crossing improvements, traffic calming improvements, safety improvements, landscape improvements, other improvements (yes = 1; no= 0). Items were summed to create the built environment activities index (0–6).
Other active travel to school activities	7	Whether any of the following activities were also occurring at the school: environmental audit and walk assessments, neighborhood improvement requests, student safety training, parent safety education, enforcement of traffic laws, direct marketing of walking and biking to school, and special events to encourage walking and biking (yes = 1; no = 0). Items were summed to create the other active travel to school activities index (0–7).
Total support activities	13	Sum of the built environment and active travel to school activities indices (0–13).

**Top implementation barriers.** From a list of 8 barriers identified from previous literature and expert input (20,22–24,26–29), we asked respondents to rank order the top 3 barriers to implementation faced by their programs. Barriers were concerns for liability and injury, distance to school, identifying and maintaining a coordinator, identifying and maintaining route leaders, lack of financial support, lack of support from school leadership, neighborhood or environmental safety, and recruiting and maintaining student participants.

**Covariates.** The participant survey captured the estimated proportion of students living within 2 miles of the school and whether school busing was available (yes or no). For schools in the United States, each school’s zip code and school name were matched with public data (41) on number of students and percentage of students eligible for free or reduced-price lunch (FRPL), with greater FRPL rates reflecting lower income areas. Rural–Urban Commuting Area (RUCA) codes (42) were identified for each US school by using zip codes, and schools with a RUCA code more than 4 were classified as rural. FRPL and RUCA information was obtained for programs only within the United States; the mean for FRPL and mode for RUCA were used to impute values for programs outside the United States.

We used descriptive statistics to present means, standard deviations, and frequencies for study variables. We presented the percentage of programs from different regions of the United States with findings from an earlier study that captured prevalence of walking school bus programs in a US representative sample (22). Differences in implementation contextual factors across income groups (based on FRPL, excluding programs outside the United States) and between programs located within the United States were assessed using linear, logistic, and multinomial regression models with Bonferroni-adjusted group comparison tests. Linear regression models were used to investigate associations of the contextual factor independent variables with the three implementation outcomes. Initial models (Model 1) tested each contextual factor in a separate model and additional models (Model 2) tested all contextual factors in the same model. We used logistic regression models to investigate associations of the implementation outcomes (independent variables) with each student outcome. Linear regression models were used to investigate associations of the implementation outcomes with the non-health benefits index. Lastly, we used descriptive statistics used to present the proportion of programs ranking each barrier in the top 3 overall and by coordinator type. For the top 2 barriers in the top 3, logistic regression models were used to investigate differences in the percentage of programs noting these barriers across coordinator types, across income groups, and by location. All models accounted for nesting of programs within respondents by using mixed-effects models and were adjusted for domestic or foreign location, rural or urban location, number of students in the school, percentages of free or reduced price lunch eligibility, percentages of students living within 2 miles of school (as perceived by the respondent), and school busing available (yes or no). Models investigating student outcomes were additionally adjusted for program coordinator type. To identify whether the findings were consistent when only US schools were considered (ie, sensitivity analyses), the same models described above were investigated in only the US schools. Analyses were conducted using SPSS Statistics 24 (IBM).

## Results

Evaluation participants were 145 key informants from 184 existing walking school bus programs. Twenty (10.9%) of the 184 participating programs involved a bike train. Twelve (8.3%) of the 145 key informants completed the survey for more than 1 school (ie, >1 program). Eighty-five (46.2%) of the key informants (ie, program coordinators) were school or district staff, 26 (141%) were parents, and 73 (39.7%) were staff from community organizations. Participating programs were from multiple regions of the United States and 6 other countries (Table 2), with 34 (18.5%) of programs from outside of the United States. At the school level (ie, all students at the school), the average estimated proportion of students living more than 2 miles from the school was 66.0% (SD = 29.5%), and 145 (78.8%) of schools had school busing. Of the US-based programs, average school size was 478 (SD = 291) students, average FRPL eligibility at the school level was 60.2% (SD = 29.5%), and 28.8% of schools were from rural areas. Rural and urban schools did not differ relative to FRPL, proportion of students living more than 2 miles from the school, and whether school busing was available. Rural schools had significantly fewer total students as compared with urban schools (mean, 347.4 vs mean, 498.9; *P* = .04).

**Table 2:** Walking School Bus Programs by Location (N = 184), 2017–2018

Location	Schools in Region, No. (%)	Percentage of Schools in Region With Walking School Bus^a^
New England (Northeast)	11 (6.0)	—
Mid-Atlantic (Northeast)	5 (2.7)	7.3
East North Central (Midwest)	36 (19.6)	—
West North Central (Midwest)	36 (19.6)	5.4
South Atlantic (South)	12 (6.5)	—
East South Central (South)	9 (4.9)	—
West South Central (South)	(0)	2.5
Mountain (West)	26 (14.1)	—
Pacific (West)	15 (8.2)	2.5
Canada	16 (8.7)	—
South Africa	7 (3.8)	—
Switzerland	5 (2.7)	—
United Kingdom	4 (2.2)	—
Denmark	1 (0.5)	—
New Zealand	1 (0.5)	—

Abbreviation: — , no data.

a Data obtained from Turner, et al (22).

Descriptive data for implementation and student outcomes are presented (Table 3). Descriptive data for the implementation contextual factors by FRPL and location (within versus outside of the United States) are presented (Table 4). Programs in low-income communities were less likely to be coordinated by a parent than programs in moderate- and high-income communities (3.2% vs 16.7% and 29.6%). Programs in low- and moderate-income communities were more likely to have external funding than those in high-income communities (71.4% and 66.7% vs 37.0%). Programs within the United States had fewer route leaders overall (mean, 4.04 vs 6.97) but more route leaders from the school (mean, 1.42 vs 0.74) and school supports (mean, 4.21 vs 3.88) than programs outside the United States.

**Table 3:** Walking School Bus Program Implementation and Student Nonhealth Outcomes (N = 184), 2017–2018

Outcomes	Schools^a^
**Implementation outcomes**	
Number of students who participate^b^	21.4 (2.92)
Percentage of all students in school who participate^c^	8.4 (13.30)
Number of trips per week, 1–10	4.4 (3.27)
Likelihood of being sustained, 1–4	3.6 (0.83)
**Improved classroom behavior**	
Yes (aware of improvement)	78 (42.40)
No (not aware of improvement)	13 (7.10)
Don’t know	85 (46.2)
**Reduced tardiness**	
Yes (aware of improvement)	39 (21.2)
No (not aware of improvement)	27 (14.7)
Don’t know	110 (59.8)
**Reduced bullying **	
Yes (aware of improvement)	36 (19.6)
No (not aware of improvement)	14 (7.6)
Don’t know	123 (68.5)
**Improved neighborhood walkability or safety**	
Yes (aware of improvement)	92 (50.0)
No (not aware of improvement)	26 (14.1)
Don’t know	57 (31.0)
**Total nonhealth benefits (0–4)**	1.33 (1.20)

a Values are no. (%), unless otherwise indicated.

b Geometric mean and standard deviation.

c US schools only.

**Table 4:** Walking School Bus Implementation Contextual Factors, by Area Income and Program

Implementation Contextual Factors	Overall Sample	Area Income^a^^,^ b (N = 150)			Location (N = 184)	
		High (No. = 27)	Moderate (No. = 60)	Low (No. = 63)	Within US (No. = 150)	Outside US (No. = 34)
**Built environment**						
Environmental supportiveness, 0–3	2.08 (1.06)	2.04 (1.06)	2.07 (1.06)	1.94 (1.13)	2.0 (1.08)	2.45 (0.85)
**Program coordination, No. (%)**						
Coordinated by school or district	85 (46.2)	13 (48.1)	25 (41.7)	37 (58.7)	75 (50.0)	10 (29.4)
Coordinated by parent	26 (14.1)	8 (29.6)^c^	10 (16.7)^d^	2 (3.2)^c^^,^ d	20 (13.3)	6 (17.6)
Coordinated by external organization	73 (39.7)	6 (22.2)	25 (41.7)	24 (38.1)	55 (36.7)	18 (52.9)
**Route leader characteristics**						
Number of route leaders	4.57 (5.14)	4.93 (4.28)	4.13 (5.21)	3.57 (2.88)	4.04 (4.20)^e^ 4.0 (4.20)	6.97 (7.80)^e^
School route leader involvement (0–3)	1.29 (1.17)	1.44 (1.19)	1.02 (1.16)^c^	1.79 (1.06)	1.4 (1.17)^e^	0.74 (0.99)^e^
External route leader involvement (0-4)	1.68 (1.24)	1.70 (1.23)	1.75 (1.26)	1.71 (1.26)	1.73 (1.25)	1.47 (1.19)
**Program funding, No. (%)**						
Any external funding	112 (60.9)	10 (37.0)^c^^,^ d	40 (66.7)	45 (71.4)^d^	95 (63.3)	17 (50.0)
**Personal supports and resources**						
School supports (0–7)	4.15 (1.95)	3.93 (2.20)	4.10 (1.82)	4.44 (1.64)	4.2 (1.82)^e^	3.88 (2.43)^e^
Parent supports (0–2)	1.36 (0.69)	1.37 (0.63)	1.37 (0.69)	1.22 (0.71)	1.3 (0.68)	1.59 (0.70)
**Support activities**						
Built environment activities (0–6)	1.35 (1.44)	0.96 (1.22)	1.52 (1.37)	1.71 (1.58)	1.50 (1.46)	0.71 (1.17)
Other active travel to school activities (0–7)	3.69 (2.19)	3.07 (1.92)	3.48 (1.85)	4.10 (2.35)	3.67 (2.11)	3.76 (2.55)
Total activities (0–13)	5.04 (2.92)	4.04 (2.47)	5.00 (2.79)	5.81 (3.35)	5.17 (3.04)	4.47 (2.30)

Abbreviation: SD, standard deviation.

a Excludes programs outside the United States.

b High income = 0% to 33% free or reduced-priced lunch eligibility (FRPL); moderate income = 33.1% to 66% FRPL; low income = 66.1% to 100% FRPL.

c,d,e Values within a row that share a common superscript are significantly different, whereas values that do not share a common superscript are not significantly different (*P* < .05).

No external funding, a greater number of route leaders, and coordination by a school or district staff member as opposed to a parent were associated with a greater number of students participating in the program (higher reach) in the fully adjusted models (Table 5). Location within the United States, a lower FRPL, greater school route leader involvement, lower external route leader involvement, external funding, and engaging in fewer support activities were related to fewer trips per week (lower dose) in the fully adjusted models. A higher FRPL, greater number of route leaders, and engaging in more built environment support activities were related to a greater likelihood of the program being sustained in the fully adjusted models.

**Table 5:** Associations Between Walking School Bus Implementation Contextual Factors and Implementation Outcomes (N = 184)

Implementation Contextual Factors	Model 1^a^^,^ d Students^c^	Model 2^b^^,^ d Students^c^	Model 1^a^ Trips Per Week^c^	Model 2^b^ Trips Per Week^c^	Model 1^a^ Likelihood of Sustainability^e^	Model 2^b^ Likelihood of Sustainability^e^
**Location and socioeconomic status**						
Within US (reference outside US)	0.03 (−0.51 to 0.57) [.91]	0.25 (−0.30 to 0.80) [.37]	−3.40 (−5.04 to −1.75) [<.01]	−2.50 (−4.12 to −0.88) [<.01]	−0.32 (−0.75 to 0.11) [.15]	−0.15 (−0.62 to 0.32) [.52]
Free or reduced-price lunch eligibility (%)	0.004 (0 to 0.01) [.27]	0.004 (0 to 0.01) [.22]	0.03 (0 to 0.04) [<.01]	0.04 (0.01 to 0.05) [<.01]	0.05 (0 to 0.01) [.06]	0.06 (0 to 0.01) [.02]
**Built environment **						
Environment supportiveness (0–3)	0.11 (−0.02 to 0.24) [.11]	0.11 (−0.02 to 0.24) [.11]	0.16 (−0.28 to 0.60) [.47]	0.08 (−0.32 to 0.48) [.71]	0.07 (−0.04 to 0.18) [.23]	0.06 (−0.05 to 0.17) [.33]
**Program coordination**						
Coordinated by school or district (ref)	[Reference]	[Reference]	[Reference]	[Reference]	[Reference]	[Reference]
Coordinated by parent	−0.52 (−1.03 to 0) [.05]	−0.58 (−1.12 to 0.02) [.04]	0.20 (−1.42 to 1.81) [.81]	−0.52 (−2.14 to 1.10) [.53]	0.09 (−0.33 to 0.51) [.69]	−0.03 (−0.50 to 0.44) [.89]
Coordinated by external organization	−0.26 (−.66 to 0.14) [.21]	−0.22 (−0.65 to 0.22) [.33]	0.27 (−0.98 to 1.52) [.67]	0.41 (−0.87 to 1.69) [.53]	−0.01 (−0.34 to 0.32) [.94]	−0.11 (−0.48 to 0.26) [.57]
**Route leader characteristics**						
Number of route leaders	0.05 (0.01 to 0.07) [.01]	0.04 (0 to 0.06) [.02]	0.06 (−0.03 to 0.16) [.23]	0.05 (−0.04 to 0.14) [.30]	0.03 (0 to 0.05) [.03]	0.03 (0 to 0.05) [.03]
School route leader involvement (0–3)	0.18 (0.03 to 0.31) [.02]	0.04 (−0.12 to 0.20) [.66]	−0.67 (−1.10 to −0.23) [<.01]	−0.95 (−1.45 to −0.45) [<.01]	−0.01 (−0.12 to 0.11) [.93]	−0.00 (−0.14 to 0.14) [.96]
External route leader involvement (0–4)	0.15 (0.02 to 0.26) [.02]	0.07 (−0.05 to 0.20) [.27]	0.40 (0 to 0.79) [.05]	0.51 (0.11 to 0.91) [.01]	0.03 (−0.07 to 0.12) [.63]	−0.01 (−0.12 to 0.10) [.86]
**Program funding**						
Any external funding	−0.40 (−0.75 to −0.04) [.03]	−0.45 (−0.82 to −0.08) [.02]	−1.54 (−2.63 to 0.45) [.01]	−1.78 (−2.87 to 0.69) [<.01]	−0.06 (−0.35 to 0.24) [.71]	−0.13 (−0.44 to 0.19) [.44]
**Personal supports and resources**						
School supports (0–7)	0.07 (−0.02 to 0.15) [.17]	−0.02 (−0.11 to 0.08) [.76]	−0.23 (−0.51 to 0.06) [.12]	−0.06 (−0.36 to 0.24) [.71]	−0.02 (−0.09 to 0.05) [.62]	−0.06 (−0.14 to 0.03) [.21]
Parent supports (0–2)	0.30 (0.06 to 0.52) [.01]	0.23 (0 to 0.47) [.05]	0.66 (−0.06 to 1.38) [.07]	0.56 (−0.15 to 1.28) [.13]	0.14 (−0.04 to 0.33) [.13]	0.11 (−0.09 to 0.32) [.29]
**Support activities**						
Built environment activities (0–6)	0.10 (0 to 0.21) [.07]	0.05 (−0.05 to 0.16) [.34]	0.31 (−0.03 to 0.66) [.08]	0.43 (0.08 to 0.76) [.01]	0.08 (−0.01 to 0.16) [.10]	0.06 (−0.03 to 0.15) [.24]
Other active travel to school activities (0–7)	0.04 (−0.04 to 0.12) [.33]	0.06 (−0.03 to 0.15) [.22]	−0.31 (−0.55 to −0.05) [.02]	−0.25 (−0.53 to 0.02) [.08]	0.06 (0 to 0.12) [.09]	0.07 (−0.01 to 0.15) [.10]
Total activities (0–13)	0.05 (0 to 0.11) [.10]	0.06 (0 to 0.12) [.08]	−0.08 (−0.26 to 0.10) [.39]	0.03 (−0.15 to 0.22) [.74]	0.05 (0 to 0.10) [.04]	0.06 (0 to 0.11) [.02]

a Adjusted for domestic and foreign status, rural and urban status, number of students in the school, % free or reduced price lunch eligibility, percentage of students living within 2 miles of school, and school busing availability.

b Additionally, adjusted for all other independent variables shown.

c Model values are β unstandardized regression coefficient (95% confidence interval [CI]) and [*P* value].

d Number of students was natural log transformed, and the regression coefficient × 100 can be interpreted as the percent change in number of students.

e Model values are odds ratio (95% confidence interval) and [*P* value].

Every additional trip per week was related to a 23% increase in the odds of experiencing a reduction in tardiness, 21% increase in the odds of experiencing a reduction in bullying, and 15% increase in the odds of experiencing improvements in neighborhood walkability and safety (Table 6). The number of trips per week was also positively associated with the nonhealth benefits index. The number of students participating in the program and odds of the program being sustained were unrelated to each perceived student outcome and the nonhealth benefits index. FRPL and program location were also unassociated with the student outcomes and nonhealth benefits.

**Table 6:** Walking School Bus Implementation Outcomes and Associations With Perceived Student Outcomes (N = 184)

Perceived Student Outcomes					
Implementation Outcomes^a^	Improved Classroom Behavior^a^	Reduced Tardiness^a^	Reduced Bullying^a^	Improved Walkability/Safety^a^	Total Nonhealth Benefits^b^ (0–4)
Number of students	1.00 (0.99 to 1.00) [.77]	1.00 (0.99 to 1.00) [.54]	1.00 (0.99 to 1.00) [.23]	1.00 (0.99 to 1.02) [.14]	0 (0 to 0) [.16]
Number of trips per week, 1–10	1.02 (0.89 to 1.16) [.72]	1.22 (1.04 to 1.43) [.01]	1.20 (1.04 to 1.39) [.01]	1.14 (1.00 to 1.30) [.03]	0.10 (0.04 to 0.16) [<.01]
Likelihood of being sustained, 1–4	0.90 (0.56 to 1.46) [.70]	0.73 (0.42 to 1.27) [.27]	0.70 (0.42 to 1.16) [.17]	1.09 (0.68 to 1.73) [.71]	−0.06 (−0.28 to 0.16) [.58]

Abbreviation: β, unstandardized regression coefficient.

a Model values are odds ratio (95% confidence interval [CI]) and [*P* value] for a yes response.

b Model values are β unstandardized regression coefficient (95% CI) and [*P* value].

Recruiting and maintaining student participants and identifying and maintaining route leaders were the 2 most commonly noted of the top 3 barriers (Table 7). Recruiting and maintaining student participants was the most commonly noted top 3 barrier for programs coordinated by schools or districts and parents, and the second and third most commonly noted top 3 barrier by programs coordinated by external organizations. Identifying and maintaining route leaders was the second and third most commonly noted top 3 barrier by programs coordinated by external organizations, and the second most commonly noted top 3 barrier by programs coordinated by schools or districts and parents. Neighborhood environment and safety (by programs coordinated by external organizations), distance to school (by programs coordinated by schools or districts), and identifying and maintaining a coordinator (by parents) were also commonly noted top 3 barriers.

**Table 7:** Walking School Bus Program Implementation Barriers Rank Ordered by Coordinator type (N = 184), 2017–2018

Barrier	Barrier Rankings in Top 3, No. (%)			
	All Programs	Coordinated by School or District	Coordinated by Parent	Coordinated by External Organization
Recruiting and maintaining student participants	93 (51)	44 (51.8)	13 (50.0)	36 (49.3)
Identifying and maintaining route leaders	82 (45)	35 (41.2)	9 (34.6)	38 (52.1)
Neighborhood environment and safety	60 (3.6)	27 (31.8)	4 (15.4)	29 (39.7)
Distance to school	54 (29.3)	32 (37.6)	3 (11.5)	19 (26.0)
Lack of financial support	52 (28.3)	24 (28.2)	7 (26.9)	21 (28.8)
Identifying and maintaining coordinator	46 (25.0)	21 (24.7)	12 (46.2)	13 (17.8)
Concerns for liability or injury	42 (22.8)	19 (22.4)	2 (7.7)	21 (28.8)
Lack of support from school leadership	40 (21.7)	16 (18.8)	5 (19.2)	19 (26.0)

## Implications for Public Health 

The primary finding was that walking school bus programs that were more likely to be sustained involved multiple route leaders and engaged in multiple active travel to school (eg, SRTS-related) activities, as compared with those that were less likely to be sustained. Findings also allowed a clearer understanding of the prevalence of different types of benefits that were perceived to have accrued from walking school bus programs. In addition to health-related benefits, 42% of programs reported perceived improvements in classroom behavior. Recruiting route leaders and engaging students to participate appeared to be the largest barriers to implementing walking school bus programs. Despite these implementation challenges, findings indicated that walking school bus programs can be implemented successfully in many different contexts and using various models. Implementation strategies that overcome barriers have potential for increasing reach and implementation rates of walking school bus programs and ultimately increasing population rates of walking to school and overall physical activity in children.

### Route leaders, personal supports, and resources

Programs with more route leaders reached more students and were more likely to report sustainability than programs with fewer route leaders. This may in part be because route leaders and other program leaders often leave a school. For example, a parent may leave the program when their child moves to middle or secondary school or a teacher may move schools, which could cause a program to fail if other leaders were not involved. Therefore, it appears important to involve multiple people in leadership roles to implement walking school bus programs and maximize program sustainability. Although the source of the route leader, whether they were school staff or from outside of the school (including parents), did not appear important to sustainability. Programs that operated more frequent walks had fewer school staff and more people from outside of the school serving as route leaders than those operating few walks. People outside the school might have been more intrinsically motivated to support walking school bus programs and, therefore, willing to commit more time to the program than those inside the school. Particularly, parent involvement appears to be important to supporting reach and dose as indicated by findings in the subsample of US schools. Although involving parents as route leaders might not always be feasible, involving them in other ways is likely to support program success.

### Built environment and other active travel-to-school activities

Engaging in a built environment, in conjunction with other active travel to school-related supports, appear to be important to supporting walking school bus sustainability and reach. Active travel to school support activities can include sidewalk and street crossing improvements, safety training, and promotional events (43). Engagement in these activities can complement walking school bus efforts, and it likely reflects a commitment at the school to support walking to school, which could explain why these activities were related to better program sustainability and reach. Although sustainability and reach benefited from both a built environment and other active travel to school activities, dose was only related to built environment activities. Although previous studies have indicated that other active travel to school-related activities, such as special events and student safety education and training activities, are likely to support increases in walking to school in general (43), integration of such activities with the walking school bus might maximize direct benefits to walking school bus programs. For example, the walking school bus can be promoted during other active travel to school-related events, and results from these activities (eg, increased pedestrian safety) can be communicated to parents with a walking school bus sign-up sheet.

### Program coordinators and existing built environment characteristics

The source of the program coordinator (ie, school staff, parent, or external organization) was unrelated to program reach, dose, and sustainability, with the exception that programs coordinated by parents (full sample) and by external organizations (US schools subsample) had a lower reach than those coordinated by school or district staff. These findings generally suggest that walking school bus programs can be implemented successfully by different types of leaders. This is a promising finding because while school- or parent-led programs may work well in some contexts, other contexts may require external leaders to organize and coordinate a program. For example, very few programs in lower-income areas were coordinated by a parent, suggesting that parent involvement may have been challenging in these contexts, perhaps due to disparities in resources that limit parents' availability. Programs appeared to find success even when there was a low level of support from the school (school supports), suggesting that walking school bus programs can be implemented successfully without strong school involvement. However, the findings related to program coordinator type and reach suggest that partnerships with schools may be important for improving reach when programs are led by parents or external organizations. Another positive finding was that programs appeared to find success even when the neighborhood environment was somewhat unsupportive of walking. This finding was likely because walking school bus programs provide safety in groups and through adult supervision, which can compensate for a somewhat unsafe environment. However, few respondents reported very low school or neighborhood environment supportiveness, so there may be a threshold for these factors that needs to be exceeded for a program to be successful that was not well captured in the range of responses in the present sample.

### External funding

Interestingly, having external funding was unrelated to sustainability and negatively related to reach and dose, though the negative associations with reach and dose were not observed in the subsample of only US schools. Walking school bus programs, while challenging to initiate, can be operated with little cost due to volunteer involvement. Funding is often primarily used for providing stipends to route leaders and to pay for coordinators’ time when they are from outside organizations, and it is possible that larger programs in the present study had internal rather than external funding. Sources of internal funding could include fundraising by the Parent Teacher Organization at the school or district allocation of funding specifically for the walking school bus.

### Neighborhood income

A positive finding was that programs in lower-income areas were more likely to have outside active travel to school funding than those in high-income areas and were reported to be more likely to be sustained. This may be in part because many SRTS efforts, and thus a higher amount of resources, are often focused in lower-income communities to support equity (43). However, the low level of parent involvement in serving as routes leaders in these communities suggests that strategies that engage parents or help to identify other sustainable sources of involvement (eg, others invested in the community) are particularly important low-income communities. Identifying other sources of route leaders may be promising because parents in low-income communities may have other high-priority obligations that limit their availability or ability to engage in leading a walking school bus program.

### Student benefits

Assessing whether the walking school bus program led to beneficial outcomes for students in this sample was challenging because objective information on such outcomes was not available. Thus, we relied on respondents to report whether they were aware of students experiencing benefits, so these findings should be interpreted with caution. The most prevalent student nonhealth benefit reported by program leaders was improved classroom behavior, reported by almost half of respondents. This was an important finding because it suggests that walking school buses can support outcomes that are important to schools and teachers, in addition to providing benefits directly to students. Highlighting such benefits may support increases in school involvement in walking school bus programs. Programs providing more frequent walks were more likely to report reductions in tardiness and bullying and improvements in neighborhood walkability and safety (15%–23% greater odds per each additional trip per week) than programs with less frequent walks. Daily walks also contribute more to overall physical activity and health promotion (4,26,44), and thus should be targeted more often by walking school bus programs, as the average number of trips per week in the present sample was less than 5. Although the number of student participants was not associated with respondent perception of nonhealth benefits, higher reach programs are likely to produce benefits for greater numbers of students and, therefore, be more effective at the population level. The lack of association between sustainability and perceived student benefits was likely due to all programs operating; if a program ended, those benefits would likely cease.

### Implementation challenges

The most challenging aspects of walking school bus implementation, as suggested by respondents’ rank order of a list of 8 barriers that were provided in the survey, were recruiting and maintaining route leaders and recruiting and maintaining student participants. Because having multiple route leaders involved appears important to sustainability but particularly difficult to obtain, based on the ranking of barriers, research is needed to identify effective implementation strategies for engaging route leaders. Programs led by parents appeared to have a slightly easier time recruiting route leaders than programs led by school staff or external organizations, based on a lower endorsement (34.6% vs. 41.2% and 52.1%) of this barrier, perhaps because parents were networked with other students’ families in their neighborhoods. This finding, paired with the finding that involving route leaders who were not school staff was related to higher dose, highlights the importance of involving parents in walking school bus programs.

This is the first study to investigate implementation factors in a large sample of walking school bus programs. Study strengths included the large number of programs assessed across the United States and internationally and a foundation in implementation science concepts and frameworks. Although extensive efforts were used to identify and recruit as many walking school bus program leaders as possible to participate in this study, our convenience sample was likely not representative of all existing walking school bus programs because of the likelihood of missed programs and participation bias, particularly for programs outside of the United States due to greater difficulty in identifying those programs. Furthermore, programs in the US midwest appeared to be overrepresented, and programs in the US northeast appeared to be underrepresented. Most study measures, including program likelihood of being sustained and perceived student benefits, relied on respondent reports. The possibility exists that these benefits were experienced by more programs but that the program leader was unaware, particularly when the program leader was not a school staff member. Pilot tests of the survey suggested, however, that walking school bus leaders are generally aware of the benefits experienced by their students and that nonschool program leaders often maintain close contact with the school and hear about benefits. Some survey respondents might have reported being aware of student benefits based on little-to-no evidence, based on social desirability bias. Reliability and criterion validity of the survey items used in the study were not assessed. Future studies are needed that follow programs longitudinally to better understand sustainability and use more sensitive measures to investigate nonhealth benefits in large samples (eg, teacher reports of classroom behavior, objective school data on tardiness). Improved studies of behavioral and academic outcomes are worthwhile because such outcomes are often most important to school decision makers. While our study uncovered key barriers to implementation, the list of barriers provided was not comprehensive; therefore, other barriers are likely to exist. Future studies should aim to capture more breadth and depth of information regarding barriers and how to overcome them (eg, best practices for recruiting and maintaining student participants and route leaders), including through the use of qualitative methods. Well-designed trials are needed to test whether strategies that address the barriers identified here result in greater program reach, dose effect, and sustainability.

Walking school bus programs are being implemented in many different contexts using various models and have potential for increasing the currently low rates of walking to school and overall physical activity in school-aged children. To improve the reach and population effects of such programs, effective strategies for engaging students, parents, and other community members are needed, as these appear to be key barriers to implementation and factors related to implementation success.
